# How have we measured trial outcomes of asthma attack treatment? A systematic review

**DOI:** 10.1183/23120541.00660-2023

**Published:** 2024-02-26

**Authors:** Imran Howell, Aleksandra Howell, Sanjay Ramakrishnan, Mona Bafadhel, Ian Pavord

**Affiliations:** 1 Respiratory Medicine Unit, Nuffield Department of Medicine, University of Oxford, Oxford, UK; 2 NIHR Oxford Biomedical Research Centre, University of Oxford, Oxford, UK; 3 School of Medical and Health Sciences, Edith Cowan University, Joondalup, Australia; 4 King’‌s Centre of Lung Health, School of Immunology and Microbial Sciences, Faculty of Life Sciences and Medicine, King’s College London, London, UK

## Abstract

**Background:**

Asthma attacks are a common problem for people with asthma and are responsible for significant healthcare costs. There is interest in a precision medicine approach to treatment. However, the choice of trial outcome measures for asthma attack treatment is hampered by the absence of a consensus on suitability. We carried out a systematic review to understand the characteristics of outcome measures used in randomised controlled trials of asthma attack treatment. Have randomised controlled trials of asthma attack treatment measured outcomes that are useful to patients and healthcare providers?

**Methods:**

The protocol was registered on PROSPERO (CRD42022311479). We searched for randomised controlled trials comparing treatments for adults with asthma attacks, published in English between 1972 and 2022 on MEDLINE, Embase and Cochrane Library databases. We recorded the outcome measures and study characteristics.

**Results:**

We identified 208 eligible randomised controlled trials from 35 countries. Trials ranged from 12 to 1109 participants, with a median of 60. The most common settings were the emergency department (n=165) and hospital admission (n=33). Only 128 studies had primary and secondary outcomes defined clearly. In those that did, 73% of primary outcomes measured change in lung function or other physiological parameters over a short period (usually <24 h). Patient-reported and healthcare utilisation outcomes were the primary outcome in 27%.

**Conclusions:**

Outcomes in randomised controlled trials of asthma attack treatment focus on short-term changes in lung function and may not capture patient-centred and economically important longer-term measures. More work is needed to investigate patient and other stakeholder preferences on core outcome sets.

## Shareable abstract

**Trials of acute asthma treatment use inconsistently defined outcome measures that focus on short-term, physiological changes. Consensus and patient-preference methods can help standardise patient-centred and economically important longer-term measures.** https://bit.ly/40XH1u0

## Introduction

Asthma is a common and important disease affecting all ages worldwide. Asthma attacks, or exacerbations, are acute deteriorations in asthma symptoms and/or lung function, which impact people’s physical activity, work, personal life and mental health [1]. They are responsible for substantial healthcare costs [2].

The asthma field has taken fledgling steps towards personalised medicine [3]. Different phenotypes of asthma can now be targeted with specific monoclonal antibody treatment to reduce asthma attacks. There is increasing evidence of heterogeneity of mechanisms driving asthma attacks, although the implications for treating asthma attacks are not completely understood [4, 5]. Current interventions for asthma attacks have not changed for many years and are potentially limited by a “one size fits all” approach, a focus on short-term outcomes, and by the administration of treatments in multiple settings (*i.e.* self-administered by the individual, prescribed in primary care or administered during an emergency/hospital care attendance [1]). There is an unmet need for targeted, safe treatments of asthma attacks that provide lasting disease stability and facilitate the goal of clinical remission [6]. Clearly and consistently defined outcome measures with appropriate timepoints are crucial to assess the efficacy of new asthma attack interventions. These outcome measures should be developed by multilateral consensus to ensure that they are relevant to people with asthma, healthcare professionals and healthcare funders [7]. An expert group previously proposed definitions of exacerbations for longer term asthma trials but standardising the outcomes for intervention trials of acute asthma was not part of their remit [8].

Our systematic review aims to identify and evaluate the outcome measures that have been used in interventions for asthma attacks to date. We will focus on the strengths and limitations of different measures and discuss how these may be adapted for future trials of asthma attack treatment.

## Methods

We prospectively registered the protocol for this systematic review on PROSPERO (CRD42022311479).

### Criteria for inclusion

We included any randomised controlled trial in humans that compared treatments for adults presenting with an asthma attack. We included any intervention or treatment compared to any control group in any setting. We included trials performed in any gender, any ethnicity and any asthma severity.

### Search strategy

We electronically searched for trials published in English between 1972 and 2022 on MEDLINE, Embase and Cochrane Library databases. The search was performed on 17 March 2022. We included conference proceedings but not study protocols. We excluded systematic reviews and meta-analyses of randomised controlled trials. We did not seek out unpublished studies. We repeated the search prior to the final analysis. The full search terms for MEDLINE and Embase are in supplementary table S1.

**TABLE 1:** Baseline characteristics of the studies included in systematic review

**Trials, n**	208
**Publication year, median (range)**	1999 (1977–2022)
**Participants per trial, median (range)**	60 (12–1109)
**Setting, n (%)**	
Emergency department	165 (79)
Hospital admission	33 (16)
Outpatient department	5 (2.5)
Intensive care	2 (1)
Pre-hospital emergency care	2 (1)
Primary care	1 (0.5)
**Region, n (%)**	
North America	93 (45)
Europe	35 (17)
Middle East	21 (10)
East Asia	17 (8)
South America	16 (7.5)
Oceania	11 (5)
South Asia	9 (4.5)
Africa	5 (2.5)
Multinational	1 (0.5)
**Blinding, n (%)**	
None	46 (22)
Single	14 (7)
Double	148 (71)
**Multicentre, n (%)**	
No	164 (79)
Yes	44 (21)
**Protocol published, n (%)**	
No	187 (90)
Yes	21 (10)^#^
**Category of intervention, n (%)**	
Nebulised therapy	62 (30)
Systemic therapy (not corticosteroid)	54 (26)
Inhaled therapy	38 (18)
Systemic corticosteroid	33 (16)
Ventilatory support/oxygenation	16 (7.5)
Patient education	3 (1.5)
Patient management pathway	2 (1)
**Primary outcome defined, n (%)**	
No	80 (38)
Yes	128 (62)

^#^: all studies with prospective protocols were published after 2010.

Title and abstract screening and study selection were performed by two review authors independently. Duplicate articles were removed. Articles were excluded if they did not meet the inclusion criteria or met exclusion criteria. Rayyan software (www.rayyan.ai) was used to record decisions and any discrepancy on study inclusion was resolved with a face-to-face meeting. If no agreement could be reached the study was excluded and recorded.

### Data extraction

Data extraction was performed by one review author (IH) and validated by a second review author (AH).

The data fields extracted from each study were year of publication, setting, number of participants, type of comparator and intervention, multicentre or single centre, presence of a defined primary outcome and outcome measures, including type, definition and timing.

### Data synthesis

The primary outcome of this systematic review was to assess the type, definition and timing of outcomes that are reported in randomised controlled trials of treatment for asthma attacks.

We used descriptive statistics to analyse the characteristics of included trials. We grouped outcomes from each trial into broad categories based on their purpose. We analysed the category, timing and definition of each outcome measure and how this related to the study setting, the year of publication and types of intervention and comparator in the trial. We performed these analyses in all studies, and in the sub-group of studies with a defined primary outcome.

We critically appraised the strengths, weaknesses and relevance of the outcome measures to patients and healthcare professionals.

## Results

### Summary of study populations and designs

**FIGURE 1:**
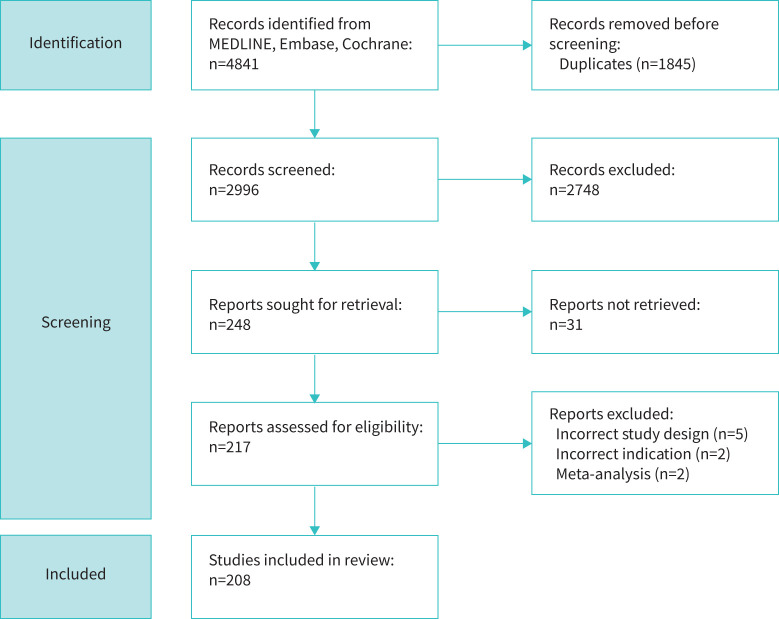
Preferred Reporting Items for Systematic Reviews and Meta-Analyses flow diagram for included studies.

The search strategy found 4841 results. 208 randomised controlled studies met the criteria for data extraction (figure 1). All studies were published between 1977 and 2022 in 35 different countries. Table 1 summarises study characteristics. We could not retrieve the full text of 31 studies after contacting the authors.

### Analysis of outcome measures

**FIGURE 2:**
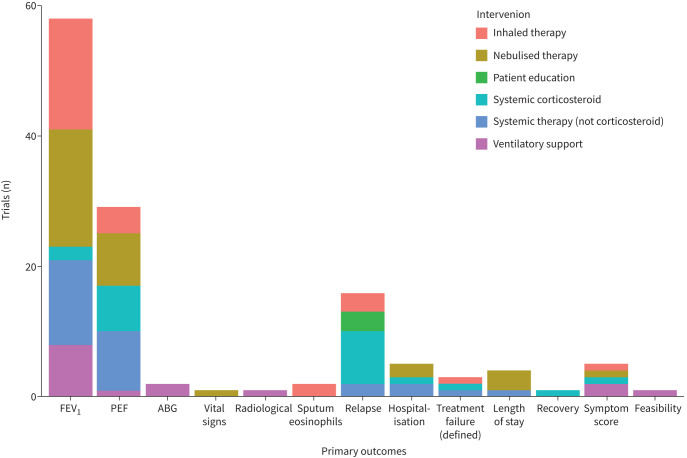
Primary outcomes reported in trials of asthma attack treatment. This stacked bar chart displays the 128 trials of asthma attack treatment between 1970 and 2022 that reported primary outcomes. The bars have been coloured to show the categories of different interventions under investigations. Outcomes have been grouped next to each other by category of physiological, healthcare utilisation, patient reported or other. Measures of lung function were the primary outcome in the majority of studies. FEV_1_: forced expiratory volume in 1 s; PEF: peak expiratory flow; ABG: arterial blood gas.

There are a variety of ways an investigator can examine the effect of a treatment for asthma attacks. These outcomes can be broadly categorised as physiological, healthcare utilisation and patient reported. We identified the categories and definitions of outcomes from 208 studies (figure 2).

### Physiological outcomes

#### Lung function

**FIGURE 3:**
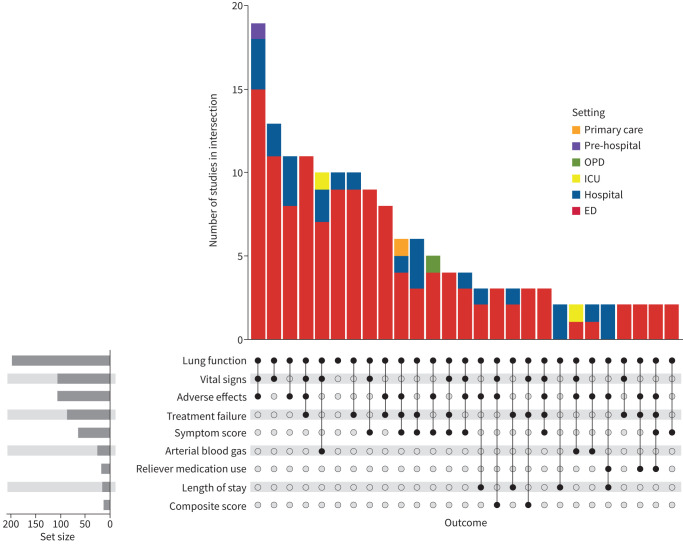
The profile of outcomes chosen for trials of asthma attack treatment. An UpSet plot demonstrating the grouping of different types of outcome measures used in trials of asthma attack treatment. In the lower panel, intersections are formed (represented by black dots connected with a line) where a trial reported those outcomes. The bar graph above is a count of the number of trials that report different intersections, coloured by setting. The bar graph on the bottom left shows the total number that each outcome was reported for all trials. Intersections where only one trial reported a specific combination of outcomes are not shown. Note that forced expiratory volume in 1 s (FEV_1_) and peak expiratory flow (PEF) have been combined into the outcome “lung function”, and types of treatment failure defined in table 2 have been grouped as “treatment failure”. The graph highlights that most trials were conducted in the emergency department (ED) setting and predominantly report physiological outcomes. OPD: outpatient department; ICU: intensive care unit.

Measures of lung function, either with forced expiratory volume in 1 s (FEV_1_) or peak expiratory flow (PEF), were the most common outcome measures. They were reported in 197 studies (95%) and made up 87 of the 128 defined primary outcomes (68%). PEF and FEV_1_ were co-reported in 34 studies (figure 3).

The timepoint for lung function measures was generally short (median 3 h).

#### Gas exchange/vital signs

25 studies reported gas exchange status measured by arterial blood gas. There was a broad mix of different interventions, with most studies based in the emergency department or hospitalised patients. The median timepoint for measuring gas exchange was 2 h. Gas exchange was selected as the primary outcome in only two studies of oxygen therapy, both in the emergency department, at 20 min and 1 h timepoints, respectively.

Oxygen saturation (through pulse oximetry), respiratory rate and pulse rate were often reported as surrogate outcomes for clinical response. 105 studies reported vital signs, although they were the primary outcome in only one trial of nebulised budesonide in the emergency department. The median timepoint for vital signs reported in these studies was 2 h.

#### Biomarkers

Three studies reported a biomarker as the primary outcome. Two studies comparing inhaled corticosteroids to other treatments used sputum eosinophils at 14 and 21 days, respectively. One study comparing nebulisation with and without noninvasive ventilation assessed radio-deposition index, radio-aerosol penetration index and pulmonary clearance at 1 h.

Five other studies reported a biomarker measurement as a secondary/exploratory outcome. These included blood eosinophil count, serum cortisol, eosinophil-derived protein and ventilation/perfusion quantification.

### Healthcare utilisation outcomes

Healthcare utilisation outcomes reflect the frequency and intensity of healthcare contact by a patient. They provide important information relevant to healthcare users, providers and policymakers [9]. This information can be related to healthcare costs and resource allocation, and disruption to a patient’s life. We identified that studies of asthma attacks tended to report more healthcare utilisation outcomes over time.

Forms of treatment failure are commonly used to judge healthcare utilisation for asthma attacks. The term is broad and inconsistently defined. It encompasses the need for further acute medical treatment, hospitalisation or unscheduled re-attendance to primary or secondary care. Many studies used outcomes that were forms of treatment failure but did not explicitly use the “treatment failure” term. We have summarised different outcomes that fall under the umbrella of treatment failure in table 2.

**TABLE 2:** The many faces of treatment failure in acute asthma trials

**Outcome reported**	**Definition**	**Median timepoint**
**Hospitalisation**	Hospital admission after a period of treatment in the ED	3 h (45 min–7 days)
**Relapse/repeat healthcare attendance**	Either one or a combination of: • unscheduled healthcare visit • repeat acute asthma treatment • hospital/ED attendance • composite of PEF, symptom score, SABA use	21 days (7 days–1 year)
**Treatment failure (defined in study)**	Composite definition: • PEF, symptom score, patient withdrawal due to uncontrolled asthma • Clinical index score, PEF, *P*_CO_2__ • Additional acute asthma treatment, hospitalisation • Intensive care admission, invasive mechanical ventilation, additional systemic corticosteroids Medication definition: • Additional systemic corticosteroids • Additional acute asthma treatment	4 h (1 h–90 days)
**Intensive care requirement**	Either one or a combination of: • ICU admission • escalation to invasive mechanical ventilation • mortality	1 day (4 h–30 days)
**Other**	Mode of baby delivery (need for emergency caesarean section)	N/A

ED: emergency department; PEF: peak expiratory flow; SABA: short-acting β-agonist; *P*_CO_2__: carbon dioxide tension; ICU: intensive care unit.

Other health utilisation outcomes measured included length of hospital stay and a health economic assessment.

#### Treatment failure

##### Defined treatment failure outcomes

There were eight studies that specifically used the term treatment failure as an outcome (three of them as a primary outcome) and provided their own definition. In three studies, treatment failure referred to a need for additional acute asthma medication. In five studies, treatment failure was a composite outcome that involved a combination of healthcare utilisation, patient-reported outcomes and physiological, medication and clinical examination parameters.

##### Hospitalisation from the emergency department

Most of the studies we identified were set in the emergency department. Hospitalisation after an intervention in the emergency department was reported in 50 studies (24%) and was the primary outcome measure in five of them. The timepoint for measuring hospitalisation from the emergency department was almost exclusively short (median 3 h). This short-term definition of hospitalisation was the only health utilisation outcome reported in 43 studies (21%).

##### Repeat healthcare attendance

Relapse or repeat hospital attendance after discharge were other commonly reported healthcare utilisation outcomes. 31 studies (15%) reported one of these outcomes, and they were a specified primary outcome in 16 studies. 25 studies were set in the emergency department, and six studies were during hospital admission. Studies reporting these outcomes had longer timepoints (median 21 days).

##### Other forms of treatment failure

Five studies specifically measured either admission to the intensive care unit, requirement of invasive mechanical ventilation or mortality. None of these was a primary outcome. Four studies tested ventilatory interventions and one study tested nebulised magnesium.

One study of nebulised magnesium therapy in pregnant women with an asthma exacerbation, set in the emergency department, measured the need for an emergency caesarean section as an outcome.

#### Length of stay or time to recovery

Length of stay in hospital was reported in 16 trials and was the primary outcome in three. They were a mix of studies testing interventions in the emergency department or during hospital admission. The definition was always based on the duration to hospital discharge.

Time to recovery at 2 weeks was the primary outcome in one trial comparing two regimens of oral steroid treatment in the emergency department. It was defined as the number of days until return to normal activity.

#### Health economics

Two studies reported health economic assessments. One study compared the total care cost per patient over 56 days post exacerbation, between ambulatory care and hospital admission. Another study reported cost per patient at 6 h after salbutamol, nebulised or inhaled with a spacer.

### Patient-reported outcome measures

#### Symptom scores

There was a variety of validated and non-validated symptom scores used in the studies (table 3). The minimum clinically important difference of these scores was rarely quoted.

**TABLE 3:** Summary of the patient reported outcome measures used in acute asthma trials

**Patient-reported outcome measure**	**Definition**
**Symptom scores**	
Modified Borg Dyspnoea Scale	12-point scale consisting of verbal and numerical descriptions of breathlessness, both at rest and during activity. The reliability and validity for the modified Borg scale has been reported many times [10].
Visual Analogue Scale (VAS)	The participant places a mark on a 100 mm long line, with anchors to indicate extremes of a symptom. The reliability and validity of VAS dyspnoea and cough have been reported [11].
Asthma Control Questionnaire (ACQ)	Well-validated, 7-point, multidimensional questionnaire assessing symptoms (five items), rescue bronchodilator use (one item), and FEV_1_ % (one item) [12]. ACQ-5 is an abridged version with just the five symptom items.
Daytime and night-time daily diary cards	Validated diary cards designed to capture the effect of asthma symptoms on daytime activities and night-time awakenings respectively [13].
Unvalidated symptom outcomes	There were numerous scores constructed for the purposes of a trial measuring symptoms including wheeze, cough, sleep, dyspnoea and sputum production. Questionnaire types included Likert scales, global symptom scores and daily diary cards.
**Quality-of-life scores**	
Asthma Quality of Life Questionnaire (AQLQ)	Validated 32-item questionnaire covering four domains using Likert scales (symptoms, activity limitation, emotional function, environmental exposure) [14].
Short-Form Health Survey (SF-36)	A questionnaire designed for use in community/outpatient settings. Consists of 36 questions reflecting eight domains of health: physical functioning, physical role, pain, general health, vitality, social function, emotional role and mental health. Reported to be reliable and valid in asthma [15].

FEV_1_: forced expiratory volume in 1 s.

**FIGURE 4:**
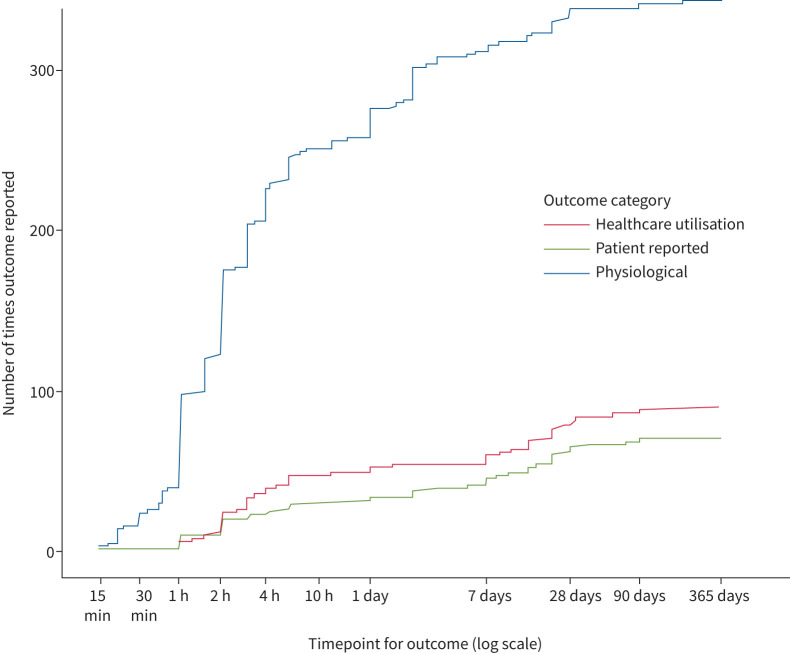
The timepoints reported for different outcome categories in trials of asthma attack treatment. A cumulative frequency graph displaying the timepoint after randomisation that categories of outcome measures were reported from all 208 trials. Outcome category is grouped as either physiological, patient-reported or healthcare utilisation (outcomes classed as “other” were not presented). The timepoints are displayed on a log scale. The graph shows that physiological outcomes were reported much more frequently that healthcare utilisation and patient-reported outcomes. Over 75% of physiological outcomes were reported at less than 24 h. A significant proportion of healthcare utilisation were also reported at under 24 h. These tended to be studies set in the emergency department reporting hospitalisation.

Symptom scores were reported in 64 studies (31%) and were the primary outcome in five of them. The median timepoint for symptoms reporting was 12 h (figure 4).

#### Quality-of-life scores

The Asthma Quality of Life Questionnaire was reported in seven studies and the Medical Outcomes Scale Short-form 36 was reported in one study. All these studies were conducted after 1997. No studies used a quality-of-life score as a primary outcome. The median time to report quality of life was 21 days.

#### Patient satisfaction

A Likert-scale satisfaction score was reported in one study comparing the treatment of asthma attacks in ambulatory care *versus* hospital admission.

### Other

#### Adverse effects

104 studies explicitly reported adverse effects. The most frequent, general adverse effects were listed in most trials. Some studies focused on reporting specific adverse effects of the intervention under investigation, including symptoms, biochemical tests and vital signs.

#### Composite clinical scores

A variety of composite scores was used as surrogate measures of asthma severity or predictors of relapse (summarised in supplementary table S2). 14 studies reported a composite score as an outcome. None of these was as a primary outcome.

#### Reliever medication use or medication adherence

Reliever medication use was reported as an outcome in 17 studies as a proxy for asthma control (never as a primary outcome). This was measured using either prescription records, number of actuations recorded on an inhaler or patient-reported use.

Patient-reported medication adherence to the investigational drug was reported in one study.

#### Pharmacokinetics

13 studies explicitly reported plasma levels of the investigational drug as an outcome. Typically, these studies tested inhaled and nebulised treatment or systemic xanthines.

## Discussion

Our methodological systematic review evaluated the outcome measures used in 208 randomised controlled trials of acute asthma since 1970. Studies were frequently small, conducted in emergency care or hospital admission settings, and lacked a defined primary outcome measure. These studies often focused on physiological outcome measures over short periods to judge interventions. We also noted significant heterogeneity between the definitions of similar types of outcome measures. Our findings suggest that the approaches taken by studies in acute asthma to date may lack generalisability, overlook other valid outcome measures, and hinder the evaluation and comparison of different interventions.

The size and setting of a clinical study bear on its external validity [16]. We observed a trend for larger studies with adequately powered primary outcomes over time; however, there were numerous interventions that have only ever been examined in underpowered trials. Small trials are perhaps understandable in the challenging setting of life-threatening asthma attacks. In terms of setting, 95% of studies we identified were conducted in the emergency department or during a hospital admission. Clearly, these are important arenas for clinical trials in acute asthma because of the high healthcare provider costs and the large impact on patients’ lives. However, the bulk of asthma attacks are treated in primary care. In the UK each year, there are approximately 2.7 million general practitioner consultations for asthma (acute and non-acute) compared with 121 000 emergency department attendances [17]. By trialling treatments for acute asthma predominantly in urgent and emergent care, the medical community has directed most interventions downstream to late, severe presentations of an attack. Consequently, outcome measures chosen for these studies tend to be short term and based around quick physiological wins.

The most common physiological outcomes we found were measures of lung function (FEV_1_ or PEF). Objective measures of airflow obstruction are particularly suited to interventions directed at reversing airflow obstruction, such as bronchodilator treatment. However, lung function correlates weakly with patient-reported recovery of symptoms and relapse [18–20]. Furthermore, there is a large degree of variability in lung function because of inconsistent testing technique and effort dependence. Therefore, the minimum clinically important difference for change in FEV_1_ and PEF has been set as 20% by expert consensus [21]. We found that many trials did not consider a minimum important difference for lung function in their design and study powering. This is problematic and limits the validity of these studies. Lung function outcomes were usually assessed over short periods. While these may be sufficient to demonstrate efficacy of interventions in the narrow context of an emergency department presentation, they almost certainly do not capture the full recovery of a person from an asthma attack.

Over time, more studies used outcomes assessing healthcare utilisation and patient-reported measures, although a significant proportion were hospitalisation from the emergency department reported at under 24 h. Outcomes assessing healthcare utilisation and patient-reported measures represent disruptive events and capture the symptoms and quality-of-life changes that are important in people’s daily lives. Healthcare utilisation outcomes are particularly important to healthcare funders because they represent additional costs to the healthcare system and wider economy [17]. We believe that the term “treatment failure” is a useful concept because it has a clear implication and embodies further strain on patients and healthcare systems. However, types and timing of treatment failure reported varied considerably, which limits comparison between trials. To rectify this, the asthma community should develop consensus definitions of treatment failure for asthma attack trials. Furthermore, developing core outcome sets, through Delphi methods augmented by patient preference research, would ensure asthma attack studies are valid to patients, healthcare professionals and funders [7].

We see potential to move the research focus beyond downstream and short-term interventions for asthma attacks. Asthma care has taken great strides in recent years with the development of targeted monoclonal antibody treatment to prevent asthma attacks, accompanied by key prognostic and theragnostic biomarkers [22, 23]. We can now contemplate asthma remission as a realistic treatment goal, and adopt a “predict and prevent” approach to achieve it by intervening earlier in appropriate patients [24, 25]. This will reduce asthma attacks but not eliminate them. People, often from deprived and underserved communities, may not engage with preventative care for a variety of reasons and have more frequent asthma attacks [26]. An asthma attack presents a golden opportunity to change the trajectory of someone’s disease because they are more receptive to health advice at this point, and the marginal cost of intervention is likely to be lower [27]. Integrated care services, or specialist rapid access airways clinics, could be used to carefully phenotype an attack and offer targeted intervention. These interventions would require a combination of patient-reported outcomes defining prompt recovery and health utilisation outcomes to ensure the person is on the path to remission.

Our systematic review has several strengths and limitations. The strengths are that it is the first systematic review to evaluate outcome measures of interventions for acute asthma, it is a prospective registered study and we used a wide timeframe to capture as many applicable trials as possible. The main limitation is that because we excluded foreign language trials, did not seek unpublished studies and many of the included studies had no published protocol, we may have been subject to selection bias of outcomes reported.

In conclusion, our study revealed that outcome measures used in interventions for asthma attacks are heterogeneous and largely focus on short-term changes in lung function. There was a paucity of patient-centred and economically important longer term outcomes. Further work is needed to investigate patient and other stakeholders’ preferences on core outcomes for asthma attack interventions.

## Supplementary material

10.1183/23120541.00660-2023.Supp1 **Please note:** supplementary material is not edited by the Editorial Office, and is uploaded as it has been supplied by the author.
Supplementary tables 00660-2023.SUPPLEMENT
Supplementary figure 00660-2023.FIGURES1
