# Supplementary material for: How have we measured trial outcomes of asthma attack treatment? A systematic review
Source: ERJ Open Res. 2024 Feb 26;10(1):00660-2023. doi: 10.1183/23120541.00660-2023 (PMC10895432; doi:10.1183/23120541.00660-2023)
Supplement: Supplementary file 1 [file 00660-2023.SUPPLEMENT.pdf]

Supplementary table 1 – Search terms used for MEDLINE and EMBASE searches

**MEDLINE**	
(asthma*adj3(acute or exacerbat* or attack* or flar*)).ti,ab.	16444
("randomi* control* trial*" or "control* clinical trial*").af. not ((exp animals/ not exp humans/) or (systematic or meta analysis).ti.)	827784
Exp Therapeutics/	4947169
Treat*.ti,ab.	6108450
3 or 4	9342195
1 and 2 and 5	1870
Limit 6 to yr="1972 – Current"	1869
**EMBASE**	
(asthma*adj3(acute or exacerbat* or attack* or flar*)).ti,ab.	25766
("randomi* control* trial*" or "control* clinical trial*").af. not ((exp animals/ not exp humans/) or (systematic or meta analysis).ti.)	1094195
Exp Therapy/	9417801
(treat* or management or efficacy).ti,ab.	9785581
3 or 4	14662759
1 and 2 and 5	2536
Limit 6 to yr="1972 – Current"	2535
**COCHRANE**	

Reference lists of all Cochrane systematic review/meta-analyses of randomised controlled trials of interventions for asthma attacks/asthma exacerbations/acute asthma	437

Supplementary table 2 – Summary of composite scores reported in acute asthma trials

**Outcome reported**	**Definition**
Fischl index	A predictive index published in 1981 for asthma attacks needing hospitalisation from the emergency department. Comprises the following presenting factors: pulse rate ≥120 per minute, respiratory rate ≥30 per minute, pulsus paradoxus ≥18 mm Hg, peak expiratory flow rate ≤120 liters per minute, moderate to severe dyspnea, accessory-muscle use, and wheezing. The index ranges from 0 to 7. An index of 4 or higher was 95% accurate in predicting the risk of relapse and 96% accurate in predicting the need for hospitalisation[28].
Unvalidated composite scores	1. Combination of patient-reported symptoms and clinical examination findings.<br>2. Global evaluation by clinicians based on the patient's symptoms and clinical status.<br>3. Clinical severity score assessing 5 components (dyspnea, wheezing, accessory muscles contraction, respiratory rate, and pulsus paradoxus), each rated on a score of 0 to 3 with a

	maximum score of 15.<br>4. Clinical score based on GINA criteria (symptoms, examination findings, lung function, vital signs) for mild, moderate and severe asthma attacks.<br>5. Clinical index based on dyspnoea, wheeze, and accessory muscle use. Graded on a 3-point scale and averaged.

Supplementary table 3 – List of references reporting each type of outcome measure

**Type of outcome measure**	**Reference number of study reporting the outcome measure**
Lung function	1, 2, 3, 4, 5, 6, 7, 8, 9, 10, 11, 12, 13, 14, 15, 16, 17, 18, 19, 20, 21, 22, 23, 24, 25, 26, 27, 28, 29, 30, 31, 32, 33, 34, 35, 36, 37, 38, 39, 40, 41, 42, 43, 44, 45, 46, 47, 48, 49, 50, 51, 52, 53, 54, 55, 56, 57, 58, 59, 60, 61, 62, 63, 64, 65, 66, 67, 68, 69, 70, 71, 72, 73, 74, 75, 76, 77, 78, 79, 80, 81, 82, 83, 84, 85, 86, 87, 88, 89, 90, 91, 92, 93, 94, 95, 96, 97, 98, 99, 100, 102, 104, 105, 106, 107, 109, 110, 111, 112, 113, 114, 115, 116, 117, 119, 120, 121, 122, 123, 124, 125, 126, 127, 128, 129, 130, 131, 132, 133, 134, 135, 136, 137, 138, 139, 140, 141, 143, 144, 145, 146, 147, 148, 149, 150, 151, 152, 153, 154, 156, 157, 158, 159, 160, 161, 162, 163, 164, 165, 166, 167, 171, 172, 173, 174, 176, 177, 178, 179, 180, 181, 182, 183, 184, 185, 186, 187, 188, 189, 190, 192, 193, 194, 195, 196, 197, 198, 199, 200, 201, 202, 203, 204, 205, 206, 207, 208
Arterial blood gas	2, 4, 6, 10, 23, 38, 50, 56, 57, 72, 76, 92, 99, 108, 117, 122, 129, 145, 150, 156, 157, 160, 170, 190, 200
Biomarker	44, 48, 57, 63, 122, 132, 138, 199

Vital signs	2, 3, 4, 5, 9, 11, 12, 13, 15, 16, 17, 18, 21, 22, 23, 26, 27, 34, 35, 37, 38, 49, 50, 57, 64, 65, 66, 67, 68, 72, 73, 74, 75, 76, 81, 82, 83, 84, 86, 87, 88, 89, 91, 92, 95, 97, 98, 99, 102, 105, 108, 109, 110, 111, 112, 115, 117, 119, 123, 124, 125, 126, 128, 129, 132, 134, 135, 136, 144, 145, 146, 147, 149, 150, 156, 157, 158, 159, 160, 161, 162, 163, 164, 165, 166, 170, 172, 175, 176, 179, 180, 181, 182, 186, 187, 189, 190, 192, 193, 194, 195, 196, 200, 201, 207
Treatment failure	1, 10, 13, 14, 16, 18, 21, 24, 28, 29, 31, 32, 39, 40, 41, 42, 43, 44, 47, 53, 59, 60, 61, 64, 65, 67, 70, 71, 72, 75, 77, 81, 84, 85, 86, 90, 94, 98, 100, 101, 103, 104, 106, 107, 108, 111, 112, 113, 118, 121, 131, 133, 138, 139, 140, 142, 143, 145, 148, 150, 151, 155, 158, 161, 162, 163, 164, 165, 166, 168, 169, 170, 173, 174, 175, 177, 180, 182, 184, 185, 187, 188, 189, 191, 195, 196
Length of stay	8, 20, 25, 38, 40, 69, 70, 101, 114, 139, 152, 167, 170, 180, 204, 208
Health economic	47, 118
Symptom score	5, 7, 8, 9, 16, 17, 19, 22, 24, 31, 32, 35, 36, 45, 47, 48, 59, 67, 69, 71, 73, 75, 82, 85, 87, 91, 93, 94, 97, 99, 104, 105, 106, 107, 108, 118, 126, 127, 128, 132, 134, 137, 138, 140, 146, 148, 150, 155, 159, 166, 168, 170, 177, 178, 183, 185, 186, 187, 189, 191, 193, 196, 199, 205
Quality of life score	17, 39, 61, 67, 118, 138, 168, 169
Patient satisfaction	118
Adverse effects	2, 3, 6, 11, 12, 13, 14, 15, 16, 17, 20, 21, 24, 26, 28, 29, 30, 33, 34, 36, 37, 41, 42, 43, 45, 46, 49, 51, 52, 54, 55, 56, 59, 60, 61, 64, 66, 67, 71, 74, 75, 78, 80, 85, 86, 87, 93, 96, 97, 98, 103, 104, 107, 109, 110, 111, 112, 114, 116, 119, 130, 131, 133, 134, 135, 136, 137, 138, 143, 146, 147, 148, 149, 153, 155, 158, 159, 160, 161, 163, 164, 165, 167, 168, 169, 170, 172, 173, 175, 176, 178, 181, 182, 183, 185, 186, 189, 192, 200, 201, 204, 206, 207, 208
Composite clinical score	3, 7, 12, 14, 18, 69, 84, 100, 116, 142, 161, 162, 176, 195

Reliever medication use or medication adherence	20, 24, 28, 45, 47, 48, 54, 72, 82, 98, 101, 114, 115, 131, 168, 169, 185
Pharmacokinetics	9, 15, 30, 74, 122, 132, 134, 141, 165, 175, 183, 200, 205
