# Supplementary figures and images for: How have we measured trial outcomes of asthma attack treatment? A systematic review
Source: ERJ Open Res. 2024 Feb 26;10(1):00660-2023. doi: 10.1183/23120541.00660-2023 (PMC10895432; doi:10.1183/23120541.00660-2023)

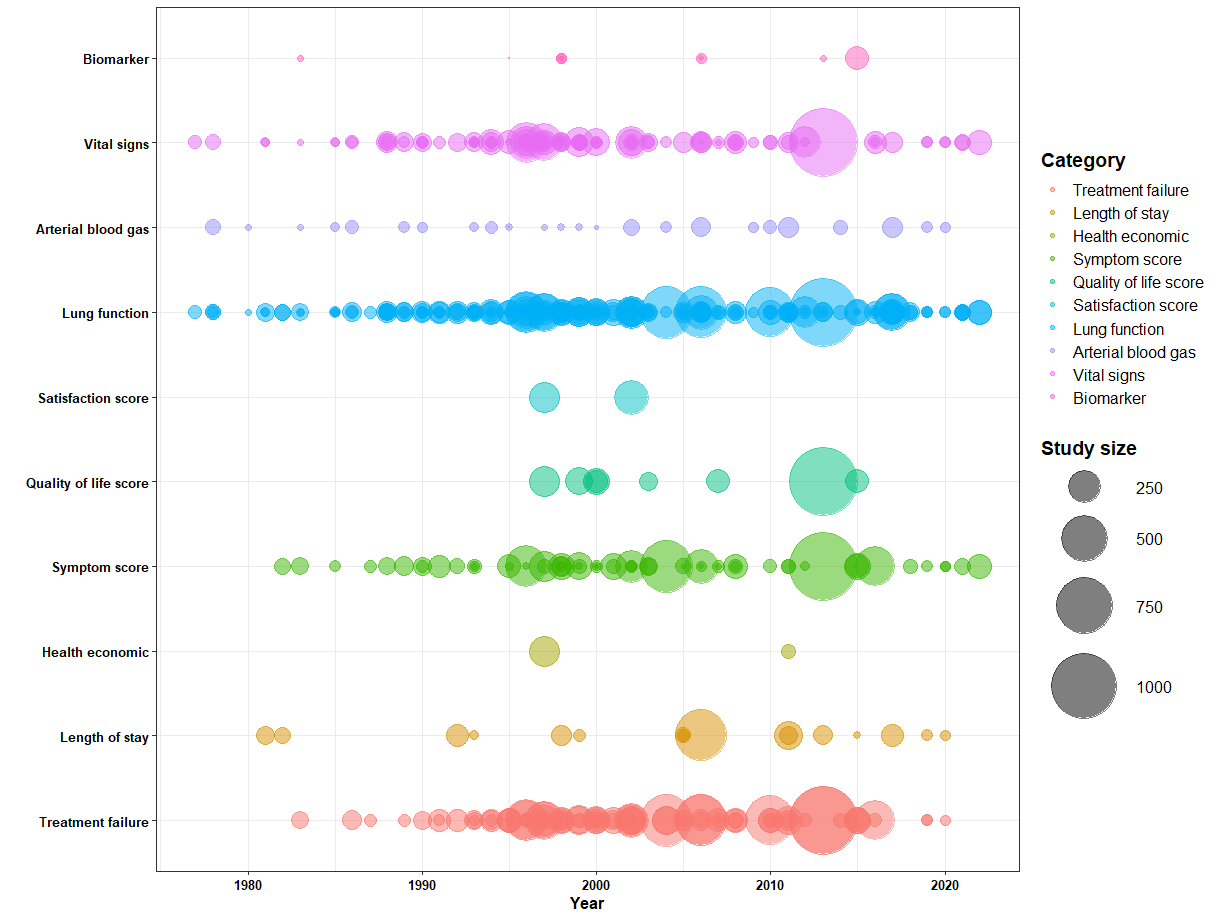

Supplement: Supplementary file 2 [file 00660-2023.FIGURES1.png]
